# Vaccinia Virus Strain MVA Expressing a Prefusion-Stabilized SARS-CoV-2 Spike Glycoprotein Induces Robust Protection and Prevents Brain Infection in Mouse and Hamster Models

**DOI:** 10.3390/vaccines11051006

**Published:** 2023-05-21

**Authors:** María M. Lorenzo, Alejandro Marín-López, Kevin Chiem, Luis Jimenez-Cabello, Irfan Ullah, Sergio Utrilla-Trigo, Eva Calvo-Pinilla, Gema Lorenzo, Sandra Moreno, Chengjin Ye, Jun-Gyu Park, Alejandro Matía, Alejandro Brun, Juana M. Sánchez-Puig, Aitor Nogales, Walther Mothes, Pradeep D. Uchil, Priti Kumar, Javier Ortego, Erol Fikrig, Luis Martinez-Sobrido, Rafael Blasco

**Affiliations:** 1 Departamento de Biotecnología, INIA CSIC, Carretera La Coruña km 7.5, E-28040 Madrid, Spain; gilsanz@inia.csic.es (M.M.L.); sandra.moreno@csic.es (S.M.); alejandro.matia@inia.csic.es (A.M.); spuig@inia.csic.es (J.M.S.-P.); 2 Department of Internal Medicine, Yale School of Medicine, New Haven, CT 06519, USA; alejandro.marinlopez@yale.edu (A.M.-L.); irfan.ullah@yale.edu (I.U.); erol.fikrig@yale.edu (E.F.); 3 Texas Biomedical Research Institute, San Antonio, TX 78227, USA; kchiem@txbiomed.org (K.C.); cye@txbiomed.org (C.Y.); jpark@txbiomed.org (J.-G.P.); priti.kumar@yale.edu (P.K.); 4 Centro de Investigación en Sanidad Animal, INIA CSIC, Carretera Valdeolmos a El Casar, Valdeolmos, E-28130 Madrid, Spain; lfj.cabello@inia.csic.es (L.J.-C.); sergio.utrilla@inia.csic.es (S.U.-T.); calvo.eva@inia.csic.es (E.C.-P.); lorenzo.gema@inia.csic.es (G.L.); brun@inia.csic.es (A.B.); nogales.aitor@inia.csic.es (A.N.); ortego@inia.csic.es (J.O.); 5 Department of Microbial Pathogenesis, Yale School of Medicine, New Haven, CT 06510, USA; walther.mothes@yale.edu (W.M.); pradeep.uchil@yale.edu (P.D.U.)

**Keywords:** poxvirus, modified vaccinia virus Ankara, vaccine, SARS-CoV-2, COVID-19, recombinant viral vectors, immunization

## Abstract

The COVID-19 pandemic has underscored the importance of swift responses and the necessity of dependable technologies for vaccine development. Our team previously developed a fast cloning system for the modified vaccinia virus Ankara (MVA) vaccine platform. In this study, we reported on the construction and preclinical testing of a recombinant MVA vaccine obtained using this system. We obtained recombinant MVA expressing the unmodified full-length SARS-CoV-2 spike (S) protein containing the D614G amino-acid substitution (MVA-Sdg) and a version expressing a modified S protein containing amino-acid substitutions designed to stabilize the protein a in a pre-fusion conformation (MVA-Spf). S protein expressed by MVA-Sdg was found to be expressed and was correctly processed and transported to the cell surface, where it efficiently produced cell–cell fusion. Version Spf, however, was not proteolytically processed, and despite being transported to the plasma membrane, it failed to induce cell–cell fusion. We assessed both vaccine candidates in prime-boost regimens in the susceptible transgenic K18-human angiotensin-converting enzyme 2 (K18-hACE2) in mice and in golden Syrian hamsters. Robust immunity and protection from disease was induced with either vaccine in both animal models. Remarkably, the MVA-Spf vaccine candidate produced higher levels of antibodies, a stronger T cell response, and a higher degree of protection from challenge. In addition, the level of SARS-CoV-2 in the brain of MVA-Spf inoculated mice was decreased to undetectable levels. Those results add to our current experience and range of vaccine vectors and technologies for developing a safe and effective COVID-19 vaccine.

## 1. Introduction

Measures for transmission control and safe and efficient vaccines have emerged as prime factors in dealing with the Coronavirus disease 2019 (COVID-19) pandemic caused by the severe acute respiratory syndrome coronavirus 2 (SARS-CoV-2). Since the spread of the disease, unprecedented efforts have been applied to develop COVID-19 vaccine candidates, resulting in numerous SARS-CoV-2-specific vaccines being evaluated in clinical trials in addition to the approved vaccines [1].

Multiple studies have identified the SARS-CoV-2 spike (S) protein as the most critical target antigen for COVID-19 vaccine development, similarly to other coronaviruses [2,3]. The trimeric S protein is a prominent immunological component of the virion surface and plays a crucial role in SARS-CoV-2 binding and entry to the host cell, mediating the interaction of the virus with the cellular receptor angiotensin-converting enzyme 2 (ACE2) and the subsequent fusion of the virus envelope with the host cell membrane. It has been clearly established that the production of S-specific antibodies that can neutralize the virus is crucial to confer protection against SARS-CoV-2 infection [4,5,6].

Diverse vaccine technologies have been explored in the race to generate suitable vaccines for COVID-19, including protein, attenuated live vectors and mRNA vaccines (for review see [7]). However, the current vaccines have limitations in providing long-term protection and may have varying levels of efficiency in preventing virus transmission [8,9,10]. One of the live attenuated vaccine vectors that can provide, alone or in combination, improved immune responses and protection is based on the poxvirus modified vaccinia Ankara (MVA) [11,12,13]. MVA constitutes an efficient vaccine for orthopoxvirus infections such as smallpox and mpox [14,15]. In addition to its use as a vaccine, and due to its proven safety profile, MVA virus recombinants have been explored as potential vaccines for diverse diseases. Since the development of MVA recombinant technology, recombinants expressing influenza A virus genes were shown to induce serum antibody levels and increased T-cell responses [16,17]. Since then, various immunization approaches using recombinant MVA have been evaluated in preclinical and clinical studies with successful results. For instance, recombinant MVA vaccines expressing HIV proteins have been tested alone or in combination with adenoviral or DNA-based vaccines to activate protective immune responses against AIDS [18,19]. MVA-vector vaccines have also been successful in vaccine development against several other infectious diseases with a global impact such as tuberculosis [20] and malaria [21]. In addition, recombinant MVA vaccines have been tested for emerging pathogens such as West Nile fever [22] and Ebola virus [23,24]. Notably, recombinant MVA-expressing Ebola virus glycoprotein is authorized in Europe as an Ebola vaccine in a combined immunization regimen [25,26,27] (https://www.ema.europa.eu/en/medicines/human/EPAR/mvabea, accessed on 18 May 2023). 

Since 2003, new beta coronaviruses causing atypical acute necrotizing pneumonia, namely the severe acute respiratory syndrome coronavirus (SARS-CoV), the Middle East respiratory syndrome coronavirus (MERS-CoV) and SARS-CoV-2, have emerged in human populations [28]. In response to the emergence of human diseases caused by beta coronaviruses such as SARS-CoV and MERS-CoV, modified vaccinia virus Ankara (MVA) has been investigated as a viral vector vaccine, with the envelope spike (S) protein being a key target for neutralizing antibodies and protection [29,30]. A recombinant MVA expressing the SARS-CoV S antigen was evaluated in mice, rabbits, and rhesus macaques, and was found to stimulate SARS-CoV-specific immune responses, including neutralizing antibodies and S-antigen-specific T cells [31,32]. Additionally, a recombinant MVA expressing the S protein of MERS-CoV was also developed and tested [33,34,35,36,37,38]. 

Not surprisingly, after the upsurge of the COVID-19 pandemic, MVA was chosen by several groups as a possible vaccine platform for disease prevention [39,40,41,42,43,44,45,46,47,48,49,50,51]. Since then, a number of different MVA-based vectors have been used to generate S-expressing MVA recombinants that have been tested in animals. So far, several studies have shown that such MVA-based vaccine candidates expressing SARS-CoV-2 S are able to confer protection against SARS-CoV-2 disease, in a way similar to that observed in coronavirus diseases caused by SARS-CoV and MERS. As a whole, those studies illustrate a variety of situations regarding the gene sequence, construction of the recombinants, inoculation protocols and characterization of the ensuing protection in different animal models. 

In this work, we generated vaccine candidates using our MVA-cloning system and showed that the resulting MVA recombinants produce a glycosylated, full-length SARS-CoV-2 S protein that is properly transported to the cell membrane. Inoculation of mice and hamsters with those recombinants induced a strong immune response that included both humoral and cell-mediated responses and was able to protect animals from lethal SARS-CoV-2 infection. We further showed that an MVA recombinant expressing a modified, prefusion-stabilized SARS-CoV-2 S has superior immunizing and protecting capacity, as tested in K18-hACE2 mice and golden Syrian hamsters. 

## 2. Materials and Methods

### 2.1. Ethics Statement

Mice were obtained from the Jackson Laboratory. Mouse experiments conducted in Spain followed the European Union guidelines 2010/63/UE and the Spanish Animal Welfare Law 32/2007. Experiments were approved by the Ethical Review Committee at the INIA-CISA and by Comunidad de Madrid (Permit number: PROEX 037/15). Animal experiments carried out at Yale University were approved by Yale Institutional Biosafety Committee (IBC) and Institutional Animal Care and Use Committees (IACUC). Protocols for IVIS imaging of SARS-CoV-2 infected animals under Animal Biosafety Level 3 (ABSL-3) conditions had been revised and approved by IACUC, IBSCYU and Yale Animal Resources Center (YARC). To minimize pain and discomfort of animals, procedures involving virus inoculation, blood drawing or imaging of animals were carried out under anesthesia.

Golden Syrian hamsters were purchased from Charles River, USA, and maintained in micro-isolator cages at Animal Biosafety Level 2 (ABSL-2) until challenge. Infections of golden Syrian hamsters with infectious SARS-CoV-2 were conducted under appropriate ABSL-3 laboratories at Texas Biomedical Research Institute, San Antonio, Texas, US. Experiments had been revised and approved by the Texas Biomedical Research Institutional Biosafety (BSC) and Institutional Animal Care and Use (IACUC) committees, under protocols BSC20-004, BSC21-026, RDC21-026 and IACUC 1722 MA, respectively.

### 2.2. Cells and Viruses

Unless specified otherwise, cells were maintained in culture medium containing 5% fetal bovine serum (FBS), 100 U/mL penicillin, 0.1 mg/mL streptomycin, and 2 mM L-glutamine and kept at 37 °C in a 5% CO_2_ atmosphere. BHK-21 cells obtained from the American Type Culture Collection (reference ATCC CCL10) were maintained in BHK-21 Glasgow minimal essential medium (Gibco BRL) containing antibiotics, glutamine and supplemented with 10% tryptose phosphate broth and 10mM HEPES. African green monkey kidney cell lines BS-C-1 (ATCC CCL-26) and Vero E6 (ATCC CRL-1586) were grown in EMEM or DMEM medium, respectively, which was supplemented with 5% FBS, 100 U/mL penicillin, 0.1 mg/mL streptomycin and 2 mM L-glutamine (Lonza, Basel, Switzerland). Human Expi293F cells (Thermo Fisher Scientific, Waltham, MA, USA) were grown in suspension following the manufacturer’s instructions.

MVA-ΔF13L is an MVA virus that has replaced the F13L coding sequence with a red fluorescent protein (dsRed) gene [52]. All vaccinia virus infections were maintained in a medium containing 2% FBS for all cell types.

Infectious SARS-CoV-2, isolate USA-WA1/2020 was obtained from the Biodefense and Emerging Infections Research Resources Repository (BEI Resources, NR-52281). Nanoluciferase (nLuc) expressing SARS-CoV-2, derived from the 2019 n-CoV/USA_WA1/2019 isolate, was generously provided by K. Plante and Pei-Yong Shi, World Reference Center for Emerging Viruses and Arboviruses, University of Texas Medical Branch. Infectious SARS-CoV-2 viruses were amplified by infecting Vero E6 cells at low multiplicity of infection (MOI of 0.01). After 72 h, cell culture supernatants were collected, clarified, aliquoted and stored at −80 °C until use. Virus infectivity was determined by plaque assay and immunostaining in Vero E6 cell monolayers. SARS-CoV-2 stocks were sequenced by using next-generation sequencing [53].

### 2.3. Design and Generation of MVAs

The isolation of recombinant MVA viruses was carried out by inserting genes into the viral genome by homologous recombination occurring between the viral genome and transfected plasmid DNA. Recombinant vaccine candidates were generated by inserting SARS-CoV-2 S gene versions in the vaccinia (strain MVA) viral genome by using a fast insertion/selection procedure [52,54]. In our constructs, S gene was placed downstream of a strong synthetic poxviral early/late promoter and inserted in an intergenic region between the viral F13L and F12L genes. The insertion does not result in the inactivation of any viral genes, thus preserving the genetic content of the original vector. The complete SARS-CoV-2 S gene from the Wuhan-Hu-1, NCBI Reference Sequence: NC_045512.2 was codon optimized to vaccinia virus codon usage (Supplementary Figure S1), and the poxviral early transcription termination signals were removed by synonym codon changes. The sequence was synthesized by Genscript Inc. (Piscataway, NJ, USA) and cloned into the transfer vector pMVA, to produce pMVA-S. The pMVA transfer plasmid contains the F13L gene of MVA, recombination flanks and a vaccinia virus early/late promoter to direct the transcription of the foreign gene [52,54].

pMVA-S was subsequently used to generate different S gene versions by codon substitutions using the Q5 Site Directed Mutagenesis kit (New England Biolabs, Ipswich, MA, USA). Mutations K986P and V987P were introduced into pMVA-S by using oligonucleotides S4800-Q5-F (TAGATTAGATccacctGAAGCTGAAGTACAAATAGATAGATTAATAAC) and S4800-Q5-R (GATAATATATCATTTAATACAGAAGATATAG), to generate plasmid pMVA-S-PP. Mutations R682S and R685S were introduced into plasmid pMVA-S-PP by using oligonucleotides S3910-Q5-F gcctctTCTGTAGCTAGCCAATCTATAATAG and S3910-Q5-R tctagaTGGAGAATTAGTTTGAGTTTGATAAG generating plasmids pMVA-S-SS-PP. Mutation D614G was introduced into plasmid pMVA-S and pMVA-S-SS-PP by using oligonucleotides S3690-Q5-F TTATATCAAGgaGTAAATTGTACTGAAGTAC and S3690-Q5-R TACAGCTACTTGATTAGAAG to generate pMVA-SDG and pMVA-S-DG-SS-PP (also termed pMVA-Spf).

Recombinant viruses MVA-S, MVA-Sdg and MVA-Spf were generated by infecting BHK-21 cells with MVAΔF13L at MOI of 0.05 followed by transfection with the corresponding transfer plasmids. Cell cultures were maintained until complete cytopathic effect and, subsequently, recombinant MVAs were selected and cloned by plaque isolation assay using standard procedures [55].

### 2.4. Western Blot

To prepare whole-cell lysates (WCL), BHK-21 cells in 6-well plates were detached from the plate and transferred to a 2 mL microcentrifuge tube and were sedimented. Cells in the pellet were lysed in 50 µL 80 mM Tris-HCl pH 6.8, 2% sodium dodecyl sulfate, 10% glycerol, 0.01% bromophenol blue and 0.71 M 2-mercaptoethanol. After lysis, 25 µL of cell extracts were analyzed by Western blot. Antibodies were diluted in phosphate-buffered saline (PBS) containing 0.05% Tween-20 and 1% nonfat dry milk for 1h at room temperature (RT). The primary antibodies used were: rabbit polyclonal anti-receptor binding domain (RBD) antibody (GeneTex, Irvine, CA, USA, ref GTX135709) diluted 1:500; mouse monoclonal anti-S antibody (GeneTex, ref GTX632604) diluted 1:10,000; rat monoclonal anti-F13 diluted 1:100. After washing three times with PBS-0.05% Tween-20, membranes were incubated with HRP-conjugated secondary antibodies for 45 min at RT. Secondary antibodies were: polyclonal goat anti-rabbit IgG (Dako–Agilent, Santa Clara, CA, USA, P0448) diluted 1:3000, polyclonal goat anti-mouse (GeneTex GTX213111) diluted 1:2000 and polyclonal rabbit anti-rat (Dako P0450) diluted 1:3000. Secondary antibodies were removed by extensive washing using PBS-0.05% Tween-20. Finally, HRP-conjugated secondary antibodies bound to membranes were detected by incubation with 1.25 mM luminol (Sigma-Aldrich, St. Louis, MO, USA), 0.2 mM p-coumaric acid (Sigma-Aldrich)-], 100 mM Tris-HCl, pH 8.5, 0.009% H_2_O_2_ for 1 min. Luminescence was recorded using a Bio-Rad Molecular Imager Chemi Doc-XRS.

### 2.5. Immunofluorescence

BHK-21 cells grown in coverslips were infected with MVA recombinants in 24-well plates. After 18 h, cell monolayers were washed with PBS and fixed by incubation with ice-cold 4% paraformaldehyde for 12 min. Those samples that were subsequently permeabilized were treated with PBS containing 0.1% Triton X-100 for 15 min at RT. All preparations were incubated with PBS-0.1 M glycine for 5 min. Subsequently, cells were incubated with primary antibodies in PBS-20% FBS for 30 min, washed with PBS and incubation with secondary antibodies diluted 1:300 in PBS-20% FBS for 30 min. Primary antibodies used included rabbit polyclonal anti-RBD (GeneTex, ref GTX135709) diluted 1:500, rat monoclonal anti-B5 diluted 1:100, an anti-mouse IgG-Alexa Fluor 488, and an anti-rat IgG-Alexa Fluor 594 (Invitrogen, Waltham, MA, USA) both diluted 1:300.

### 2.6. Protein Expression and Purification

Expi293F cells were grown to a density of 1 × 10^6^ cells/mL at 37 °C with 8% CO_2_ with regular agitation (150 rpm). Cells were transfected with a plasmid encoding SARS-CoV-2 RBD [56] using ExpiFectamine 293 transfection reagent, following the manufacturer’s instructions (Thermo Fisher Scientific). At day 4 post-transfection, medium from cell cultures were clarified by low-speed centrifugation. Recombinant RBD was purified by Ni-NTA agarose resin (Qiagen, Hilden, Germany), desalted and concentrated using Amicon columns (Millipore, Burlington, MA, USA) Purified SARS-CoV-2 S RBD was snap-frozen at liquid nitrogen and stored at −80 °C until further use. Protein content was confirmed as one protein of the expected size after SDS-PAGE analysis. Proteins were quantified by Pierce protein BCA assay kit (Thermo Fisher Scientific).

### 2.7. Mice Immunization and SARS-CoV-2 Challenge

Groups of 8–10 weeks old BALB/c mice (n = 5) were intraperitoneally, intramuscularly or subcutaneously immunized at 0 and 21 days with 10^7^ plaque forming units (PFU) of MVA or MVA recombinants MVA-Sdg, MVA-Spf or MVA. Intraperitoneally immunized mice were bled one and two weeks after prime, and two weeks after boost immunizations to determine IgM and IgG against SARS-CoV-2 S protein and the presence of neutralizing SARS-CoV-2 antibodies. Intramuscular and subcutaneously immunized mice were bled two weeks post-boost to determine IgG titers against SARS-CoV-2 S protein. Neutralizing titers were determined against infectious SARS-CoV-2 in a plaque reduction assay.

Groups of 8–10 weeks old C57BL/6 (B6) mice (n = 4) were intramuscularly immunized at 0 and 21 days with 10^7^ PFU of MVA or MVA recombinants. All animals were euthanized at day 15 post-boost, and their blood and spleens were harvested to analyze specific cellular and humoral immune responses.

Groups of 8–10 week old male and female K18-hACE2 mice were intramuscularly immunized at 0 and 21 days with 10^7^ PFU of MVA-Sdg, MVA-Spf [1] or MVA. Two weeks after the second immunization, all animals were bled to determine serum antibodies against SARS-CoV-2 S protein, and were challenged by intranasal inoculation of 10^5^ PFU of 2019n-CoV/USA_WA1/2019 isolate of SARS-CoV-2 expressing nLuc in 30 μL volume of sterile PBS, under anesthesia. Daily recordings of body weight were taken, with the initial body weight set as 100%. In survival experiments, mice were observed every 24 h and lethargic/moribund mice or mice with more than 20% loss of their initial body weight were humanely euthanized and considered to have succumbed to infection for survival plots.

### 2.8. Enzyme-Linked Immunosorbent Assay (ELISA) to SARS-CoV-2 S

Indirect ELISA was used to determine specific anti-S antibody levels in sera from immunized mice. Nunc 96-well MaxiSorp plates (Thermo Fisher Scientific) were coated with 150 ng/well of purified RBD by overnight incubation at 4 °C. Coated wells were subsequently blocked by incubation with PBS containing 0.1% Tween 20 (PBST) and 3% dried milk for 1 h at RT, and then washed three times with PBST. For antibody determination, sera were diluted in PBST 1% dried milk and incubated in the wells for 1h at RT. After three washes anti-mouse IgG-HRP secondary antibody (Dako cat. number P0447) diluted 1:2000 in PBST or anti-mouse IgG-HRP secondary antibody (cat. number AP128P, Sigma-Aldrich, St. Louis, MO, USA) diluted 1:1000 was added to the wells and incubated for 1 h at RT. After washing three times with PBST, the reaction was developed with 50 μL of substrate solution TMB (Sigma-Aldrich) and finalized by addition of the same volume of 3 NH_2_SO_4_. Results were expressed as optical density (OD) measured at 450 nm.

### 2.9. Ex Vivo Flow Cytometric Analysis

To evaluate the induced cellular immune response in mice splenocytes were collected and analyzed by intracellular cytokine staining (ICS) assay. For this, four mice immunized with MVA-Sdg, MVA-Spf or MVA were euthanized at day 15 post-immunization and their spleens harvested. A total of 10^6^ splenocytes were stimulated with 0.6 nmol (approximately 1 μg/peptide) of a peptide pool of the S protein (The PepTivator^®^ Peptide Pools, Miltenyi Biotec, Bergisch Gladbach, Germany) composed by peptides of 15 amino acid length with 11 amino acid overlapping previously selected for efficient in vitro stimulation of antigen-specific CD4+ and CD8+ Tcells. During a 6 h stimulation period cells were maintained in RPMI 1640 containing 10% FCS and 5 mg/mL brefeldin A to increase intracellular accumulation of IFN-γ. After the stimulation period, cells were washed and stained for surface markers. Finally, cells were fixed, permeabilized and stained intracellularly using appropriate fluorochromes. Antibody-fluorochrome conjugates CD3-APC, CD4-FITC, CD8-PerCP and IFN-γ-PE were used for the analysis of adaptive immune responses. Those antibody conjugates were purchased from BioLegend (San Diego, CA, USA). Data were acquired by FACS analysis on a Cube 8 (Sysmex, Kobe, Japan) and were analyzed with FlowJo software.

### 2.10. Plaque Reduction Neutralization (PRNT) Assay

To quantitate neutralization activity in sera, a PRNT assay for SARS-CoV-2 was performed as previously described [57]. Experiments were conducted with 1200 PFU/well of a recombinant SARS-CoV-2 USA-WA1/2020 expressing nLuc. One day prior to infection, Vero E6 cells were seeded in a 96 well flat bottom plate at a density of 2 × 10^4^ cells per well and incubated overnight at 37 °C under 5% CO_2_ to allow for cell adherence. The following day, mouse sera (heat inactivated for 1 h at 56 °C) were serially diluted in a separate 96 well culture plate using DMEM supplemented with 100 U/mL penicillin, 100 mg/mL streptomycin, 10 mM HEPES, 0.3 mg/mL L-Glutamine, 0.12% sodium bicarbonate, 10% FBS (all from Thermo Fisher Scientific), in a Biosafety Level 3 (BSL3) laboratory. Then a dilution of infectious SARS-CoV-2 USA-WA1/2020 was prepared in DMEM containing 10% FBS. Equal volumes of infectious virus (600 PFU/assay) and sera dilutions were mixed and incubated for 1 h at RT. After incubation, culture medium was removed from the 96 well plates and virus: sera mixtures were added on the cell monolayers. After 1 h incubation at 37 °C, virus: sera mixtures were removed from wells without disrupting the cell monolayers. Then, 100 μL of each serum dilution was added to its respective well in addition to an equal volume of DMEM containing 10% FBS and was further incubated for 72 h. Subsequently, media was then removed and replaced with 10% formaldehyde for 24 h for fixation. Finally, formaldehyde solution was removed from wells and cells were stained by addition of 50 μL of crystal violet solution for visualization. NT_50_ titers were calculated as the reciprocal (log10) of the highest serum dilution neutralizing 50% of the control virus input.

Neutralization titers were alternatively determined by using a Vesicular Stomatitis Virus (VSV) pseudotyped with SARS-CoV-2 S glycoprotein lacking the 18 C-terminal amino acids located in the cytoplasmic domain [58], using a modified VSV pseudotyping protocol (M. Lorenzo and R. Blasco, manuscript in preparation).

### 2.11. Bioluminescence Imaging (BLI) of SARS-CoV-2 Infection

The imaging procedure was conducted using IVIS Spectrum^®^ 985 in a Perkin Elmer XIC-3 animal isolation chamber. Before imaging, mice were anesthetized with isoflurane inhalation using the XGI-8 Gas Anesthesia System and administered with 100 μL of nLuc substrate, furimazine (diluted 1:40 in endotoxin-free PBS), retroorbitally before imaging. The mice were placed into the pre-saturated XIC-3 chamber and imaged in both dorsal and ventral positions. After euthanasia and necropsy, the animals were imaged again by spreading additional 200 μL of substrate solution onto exposed intact organs. Infected areas of interest were identified through whole-body imaging after necropsy. For additional imaging, positive areas were isolated, washed in PBS, and placed onto a clear plastic plate. Additional droplets of furimazine solution in PBS (1:40) were added to the organs, which were then soaked in substrate solution for 1-2 min before BLI. The images were acquired and analyzed using the Living Image v4.7.3 software package from the manufacturer. Photon flux was measured as luminescent radiance (*p*/s/cm^2^/sr). Luminescent signals were considered background during luminescent threshold selection for image display when minimum threshold levels resulted in displayed radiance of areas with no tissues or tissues containing known uninfected regions. In order to determine virus spread patterns, image sequences were acquired for several consecutive days following intranasal administration of infectious, nLuc-expressing SARS-CoV-2.

### 2.12. Measurement of Viral Burden and Analysis of Signature Inflammatory Cytokines mRNA

Organs analyzed (lung and brain) were collected, weighted, and homogenized in 0.8 mL of complete RPMI media containing 100 U/mL penicillin, 0.1 mg/mL-streptomycin and homogenized with a BeadBug 6 homogenizer (Benchmark Scientific, TEquipment Inc. Sayreville, NJ, USA) in 2 mL centrifuge tubes containing 1.5 mm Zirconium beads. Virus titers were measured by plaque assay or estimated by quantitative real-time PCR (qRT-PCR). RNA transcripts for inflammatory cytokines were also measured by qRT-PCR. First, total RNA was extracted from 100 μL of the homogenized tissues using TRIzol reagent (Invitrogen) and purified using NucleoSpin RNA columns (Macherey-Nagel, Düren, Germany), reverse transcribed with iScript advanced cDNA kit (Bio-Rad, Hercules, CA, USA, Cat#1725036). For determining copies of SARS-CoV-2 N gene RNA, qRT-PCR analysis was performed using SYBR Green Real-time PCR assay, following the manufacturer’s instructions. Viral titers in clarified tissue homogenates were quantitated using standard plaque assay. Briefly, the 4 × 10^5^ Vero-E6 cells were seeded on 12-well plate. Cells were infected 24 h after seeding with 100 μL of serially diluted clarified tissue homogenates. After 1 h viral adsorption, cells were overlaid with 1 mL of pre-warmed DMEM medium containing 0.6% Avicel (RC-5811059 FMC BioPolymer, Philadelphia, PA, USA). Plaques were visualized 3 days post infection by fixing in 10% formaldehyde overnight at RT followed by staining with 1% crystal violet solution (Sigma-Aldrich) for 20 min.

### 2.13. Experiments in Golden Syrian Hamsters

Protection experiments were conducted using four-week-old animals essentially as previously described [53,59,60]. To evaluate in vivo safety of the recombinant MVA, hamsters (n = 4/group) were sedated in an isoflurane chamber and vaccinated intranasally with 1 × 10^7^ PFU of MVA-Sdg, MVA-Spf, MVA, or unvaccinated with PBS, using a total volume of 100 μL. Hamsters were vaccinated using a prime and boost regimen, with animals receiving the boost vaccination 3 weeks after prime vaccination. After prime and boost vaccination, hamsters were monitored for morbidity (changes in body weight and clinical signs of infection) and mortality (survival) daily for 21 (prime) or 36 (booster) days. Sera were collected one day before prime vaccination, at 21 days after prime vaccination, and one day before challenge with SARS-CoV-2. For viral challenge, hamsters were sedated with isoflurane and infected by the intranasal route with 2 × 10^5^ PFU of SARS-CoV-2 in a volume of 100 μL. At days 2 and 4 post-challenge, hamsters were humanely sacrificed, and nasal turbinates and lungs were collected for pathology and viral titration. Half of the organ material were homogenized in PBS using a Precellys tissue homogenizer (Bertin Instruments, Montigny-le-Bretonneux, France) and clarified by centrifugation at 21,500× *g* for 5 min before virus titration by plaque assay. The other half of the organs were kept in 10% neutral buffered formalin for pathology observation. Lung samples were photographed to observe any detectable lesions, and the resulting images were analyzed for macroscopic pathology scores using NIH ImageJ software. This involved assessing the distribution of pathological lesions, including consolidation, congestion, and pneumonic lesions, and calculating their respective areas as a percentage of the total lung surface area.

### 2.14. Statistical Analysis

Data were analyzed using GraphPad Prism software version 8.0.1 (GraphPad Software, San Diego, CA, USA). For survival experiments, data from each immunized group were compared using log-rank test. Data from the IgG and IgM ELISA, virus neutralization and ICS assays, as well as viraemia levels, cytokine expression levels and were analyzed by Kruskal–Wallis test. *p*-values lower than 0.05 were considered significant in all cases.

## 3. Results

### 3.1. Isolation of MVA Recombinants Expressing SARS-CoV-2 S

Recombinant MVA viruses expressing three different versions of the S gene were generated. MVA-S contains the complete gene for the original reference isolate (Wuhan-Hu-1, NCBI Reference Sequence: NC_045512.2). A modified version involved a sequence variant (Sdg) incorporating the D614G mutation that became prevalent at the beginning of the pandemic and is known to increase virus infectivity in humans [61]. Finally, we introduced a set of mutations in the Sdg backbone to make a version stabilized in the pre-fusion conformation. Those included two amino-acid substitutions (K986P and V987P) that were first tested in the homologous MERS coronavirus and are known to prevent the conformational change of the protein, keeping it in its prefusion state [62]. Additionally, to block the proteolytical maturation of the S protein, two additional amino-acid substitutions (R682S and R685S) located around the proteolytic cleavage site [63] were introduced. The construct containing those four mutations was termed Spf (“pre-fusion”).

### 3.2. S Protein Expression by MVA Recombinants

**Figure 1:**
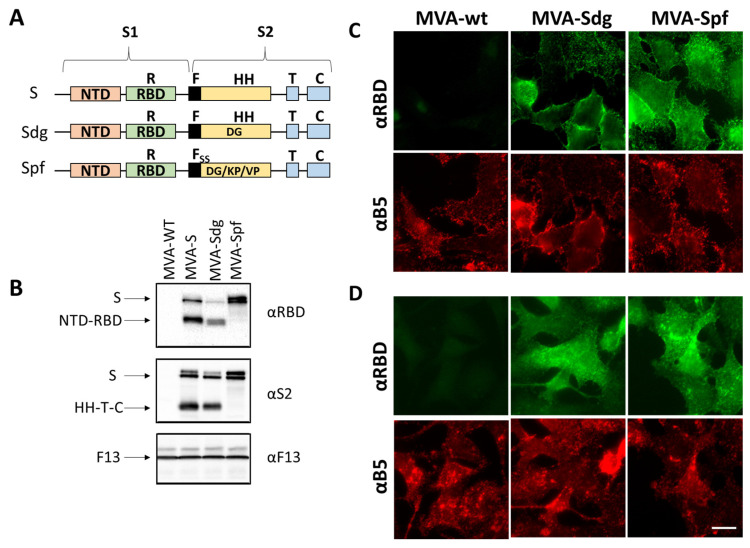
MVA recombinants expressing SARS-CoV-2 S protein. (**A**) schematic representation of the three expressed versions of the S protein. S corresponds to the original SARS-CoV-2 Wuhan-Hu-1 NC_045512.2 sequence. Sdg harbors a mutation for amino acid change D614G in the S2 domain. Version Spf contains, in addition to the D614G mutations, four extra mutations (R682S, R685S, K986P and V987P) to prevent maturation and the conformational change of the protein. Domains of the S protein are indicated: NTD, N-terminal domain; RBD, receptor binding domain; F, fusion peptide; HH, heptad repeats; T, transmembrane domain; C, cytoplasmic tail: (**B**) Western blot analysis of cells infected with recombinant MVAs. The unmodified MVA virus, and the recombinants MVA-S, MVA-Sdg and MVA-Spf were used to infect BHK-21 cells. Cell extracts were prepared at 24 h post-infection and analyzed using antibodies directed to the RBD domain or to the S2 portion of the S protein. As an infection control, Vaccinia virus F13 protein was detected using a specific rat monoclonal antibody; and (**C**,**D**) subcellular location of S protein by widefield microscopy. BHK cells were infected for 18h and stained with specific antibodies under non-permeabilizing (**C**) or permeabilizing (**D**) conditions. A specific antibody against the RBD domain was used to label the extracellular domain of the S protein. An antibody against the B5 transmembrane protein of the vaccinia virus was used as control. Scale bar, 10 μm.

After the generation of the different recombinant MVA viruses, expression of the different S proteins was assessed by Western blot and immunofluorescence in infected BHK-21 cells. Western blot with anti-RBD and anti-S2 antibodies revealed several protein products in MVA-S- and MVA-Sdg-infected cell extracts (Figure 1). The larger forms detected (180–200 kDa) were compatible with the size of the complete protein and different glycosylation states. In addition, we detected smaller forms, with an apparent mass between 90–120 kDa, that were compatible with the products (S1 and S2) generated by proteolytic cleavage in the region of the fusion peptide. Notably, the smaller forms were not present in MVA-Spf-infected cell extracts, confirming that the introduced mutations effectively prevented the proteolytic cleavage between the S1 and S2 domains in the S protein.

To further characterize the expression and subcellular location of the heterologous proteins, immunofluorescence assays were carried out. Indirect immunofluorescence on fixed and permeabilized cells produced an intracellular pattern compatible with endoplasmatic reticulum, Golgi complex and plasma membrane staining (Figure 1D). Cell-surface expression of the S protein was further confirmed by antibody staining on non-permeabilizing conditions both in MVA-Sdg and MVA-Spf infected cells (Figure 1C). Overall, these results indicated correct expression and transport of the S proteins expressed by MVA recombinants.

### 3.3. Fusion Phenotype in Cells Infected by MVA Recombinants

**Figure 2:**
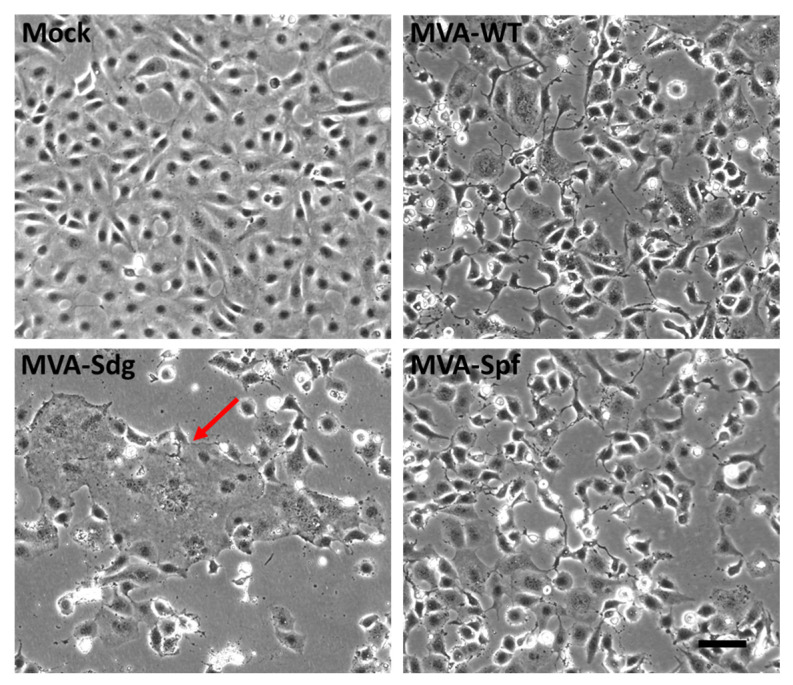
Cell–cell fusion activity in BSC-1 cells infected with MVA virus recombinants. BSC-1 cells were infected with the indicated MVA recombinants at a MOI of 3 and incubated for 18 h. Note the large syncytia (red arrow) caused by infection with MVA-Sdg and their absence in cells infected with MVA-Spf expressing the pre-fusion stabilized form of the S glycoprotein. Scale bar, 100 μm.

Mature SARS-CoV-2 S protein mediates the entry of the virus in the target cells through fusion of the viral membrane with the plasma membrane. This activity can produce cell–cell fusion between cells expressing an active S protein and the SARS-CoV-2 virus receptor (ACE2). We used this membrane fusion activity to check the integrity of the S proteins being expressed by the MVA recombinants and to confirm the predicted effect of the introduced mutations. We observed that BSC-1 cells can fuse as a result of SARS-CoV-2 expression, presumably because they display ACE-2 in their surfaces. Thus, BSC-1 cells were infected at a high multiplicity of infection with the different MVA recombinants and monitored for cell fusion. At 18 h post-infection, MVA-Sdg caused massive fusion of BSC-1 cells, producing large syncytia (Figure 2). In contrast, cell fusion activity could not be detected in cells infected with the unmodified MVA virus, indicating that cell fusion was specifically caused by the SARS-CoV-2 S protein. Interestingly, expression of the prefusion-stabilized S protein by the MVA-Spf virus did not induce detectable cellular fusion, indicating that the inserted mutations are not only impeding the proteolytic cleavage but also preventing the SARS-CoV-2 S protein-mediated fusion activity.

### 3.4. Induction of Antibody Responses in BALB/c Mice

**Figure 3:**
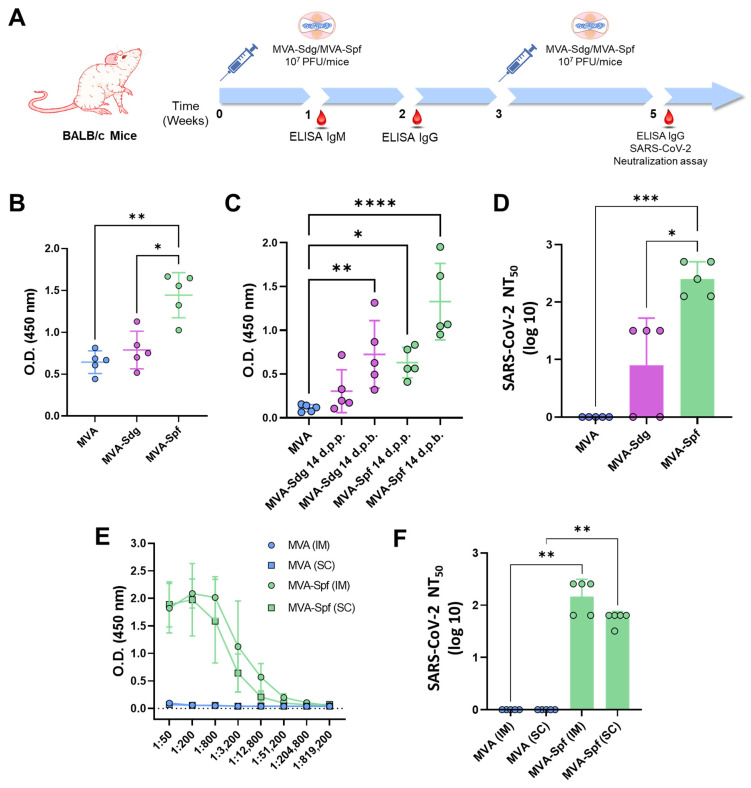
Humoral immune responses in immunized BALB/c mice: (**A**) immunization and bleeding schedule. BALB/c mice were (**B**–**D**) intraperitoneally injected with two doses of 10^7^ PFU of MVA-Sdg or MVA-Spf. A group immunized with MVA was included as control. Mice were bled at the indicated time-points; (**B**) IgM titers obtained by ELISA to the S-RBD domain with the sera (dilution 1:200) of the vaccinated animals at 7 days post-inoculation; (**C**) IgG levels specific to the RBD domain of the S glycoprotein at 14 days post-prime (d.p.p.) or boost (d.p.b) (sera dilution 1:200). Points represent individual values for each animal. Mean values for each group and standard deviations are represented. Asterisks indicate significant differences between groups (* *p* < 0.05; ** *p* < 0.0332, **** *p* < 0.0001) (Kruskal–Wallis test); (**D**) Serum neutralizing antibody titers 14 days after the second inoculation of the recombinant MVA. (* *p* < 0.05; *** *p* < 0.001) (Kruskal–Wallis test). (**E**,**F**) Comparison of the intramuscular (IM) and subcutaneous (SC) inoculation routes. BALB/c mice were intramuscular or subcutaneously immunized with two doses of 10^7^ PFU of MVA-Spf or MVA (control). Points represent individual values for each animal, and bars and error bars represent the mean values of each group and SD, respectively; (**E**) level of RBD-specific antibodies at 14 days post boost (d.p.b.) as determined by ELISA. Mouse sera were four-fold diluted starting from 1:50. Points represent mean values for each group and error bars represent SD; and (**F**) level of neutralizing antibodies 14 d.p.b in PRNT assays using infectious SARS-CoV-2. Bars and error bars indicate the mean values of each group and SD, respectively. Points represent individual values for each mouse. Asterisks denote significant differences between groups (** *p* < 0.0332, Kruskal–Wallis test).

Several works have shown that the expression of heterologous antigens downstream of the F13L gene in the MVA genome leads to the induction of potent immune responses against the specific antigens in immunized animals. To assess the induction of a humoral immune response by the MVA vaccine candidates, groups of BALB/c mice (n = 5) were intraperitoneally inoculated with two doses of 10^7^ PFU of MVA, MVA-Sdg or MVA-Spf, in a three-week-interval. Serum was harvested from the whole blood of all mice 7- and 14-days post-prime (d.p.p.), and 14 days post-boost (d.p.b.), and total IgM and IgG titers were determined. Specific antibodies to the S RBD could be detected in all mice immunized with MVA-Sdg or MVA-Spf (Figure 3A,B). Significant enhancement in the titers of anti-RBD IgM and IgG in groups immunized with MVA-Spf was evident when compared with those immunized with MVA-Sdg (Figure 3B,C). Virus-neutralizing antibodies were detected in all mice immunized with MVA-Spf, and in three out of five mice immunized with MVA-Sdg (Figure 3D). Importantly, SARS-CoV-2-neutralizing antibody titers in serum were significantly higher (*p*-value = 0.008) in animals immunized with MVA-Spf when compared to mice immunized with MVA-Sdg, indicating the superior ability of MVA expressing the pre-fusion stabilized SARS-CoV-2 S protein in eliciting a SARS-CoV-2-neutralizing humoral response.

Next, we wondered whether the inoculation route could influence the immunogenicity of our recombinant MVA-based vaccine candidates. Intraperitoneal injection of mice is often used to evaluate potency/immunogenicity of MVA vaccine candidates. However, intramuscular (IM) or subcutaneous (SC) injection routes are preferred for human use and widely accepted for vaccinia MVA vaccine administration. To test whether these alternative routes were functional for our vaccine candidates, we evaluated the immunogenicity of MVA-Spf when administered by either subcutaneous or intramuscular inoculation routes. Significant anti-RBD IgG titers were detected in all MVA-Spf-immunized mice, independently of the administration route (Figure 3E). Virus-neutralizing antibodies were also induced, reaching slightly higher titers after intramuscular immunization compared to those obtained by the subcutaneous route (Figure 3F). Based on these results, intramuscular injection was chosen as the preferred route for administration in subsequent experiments.

### 3.5. Induction of Humoral and Cellular Responses in C57BL6 (B6) Mice

**Figure 4:**
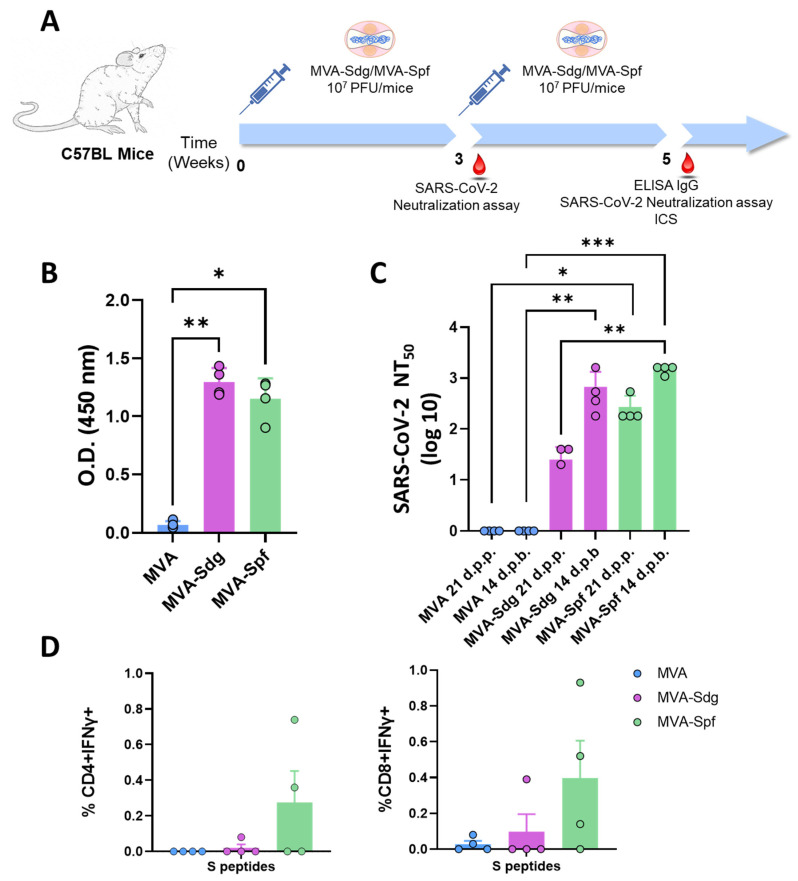
Evaluation of immunogenicity of MVA-based vaccine candidates in B6 mice: (**A**) immunization schedule. Animals were immunized intramuscularly with two doses of 10^7^ PFUs in a three-week interval. Mice were bled 21 d.p.p. and 14 d.p.b to check antibody induction. Two weeks post-boost, animals were sacrificed, and spleen cells isolated to analyze the cellular immune response; (**B**) IgG levels specific to the RBD domain of the S glycoprotein at 14 d.p.b (sera dilution 1:200). Asterisks denote significant differences between groups (* *p* < 0.05; ** *p* < 0.0332) (Kruskal–Wallis test); (**C**) detection of SARS-CoV-2-neutralizing antibodies in sera at 21 d.p.p. and 15 d.p.b. Asterisks denote significant differences between groups (* *p* < 0.05; ** *p* < 0.0332; *** *p* < 0.001) (Kruskal–Wallis test); and (**D**) cellular immune responses against the S protein of SARS-CoV-2 in immunized mice. Percentage of CD4+IFN-γ+ and CD8+IFN-γ+ T cells after stimulation with a panel of peptides spanning the S protein. Bars and error bars denote mean values and SD, respectively, for each group. Points represent individual values for each mouse. No significant differences between immunized and control mice were found (Kruskal–Wallis test).

The ability of the vaccine candidates to induce an S-specific cellular immune response was also investigated. To that end, groups of B6 mice (n = 4) were intramuscularly immunized following a prime-boost strategy with 10^7^ PFU of MVA-Sdg, MVA-Spf or MVA control, in a three-week interval. Serum from whole blood was harvested at 21 d.p.p., and at 14 d.p.b., for the analysis of the humoral immune response (Figure 4). Two weeks after the second inoculation, spleen and serum were harvested from mice and both humoral and cellular responses were analyzed. As with the immunized BALB/c mice, inoculation of our vaccine candidates resulted in significant virus neutralization antibody titers compared with MVA-inoculated B6 mice. Importantly, after the first dose of MVA-Spf, high neutralizing antibody titers were detected, which were higher in animals immunized with MVA-Spf compared to those immunized with MVA-Sdg. As in the previous experiments, boost inoculation induced enhanced antibody neutralization titers. As in the case of BALB/c mice, neutralizing antibody titers in serum were significantly higher in B6 mice immunized with MVA-Spf compared to mice immunized with MVA-Sdg, indicating the superior immunogenicity of MVA-Spf.

Splenic T-cell responses measured by intracellular cytokine staining (ICS) were detected against peptides spanning the full length of the S protein. After stimulation, splenocytes from animals vaccinated with MVA-Spf showed a moderate (non-significant) increase of CD8+ IFN-γ+ and CD4+ IFN-γ+ levels compared to the MVA (control) and MVA-Sdg immunization groups (Figure 4D), which indicates a more robust cell-mediated response after immunization with MVA-Spf.

### 3.6. Immunogenicity and Protection Efficacy of Recombinant MVAs in K18-hACE2 Mice

#### 3.6.1. Humoral Response in K18-hACE2 Mice

**Figure 5:**
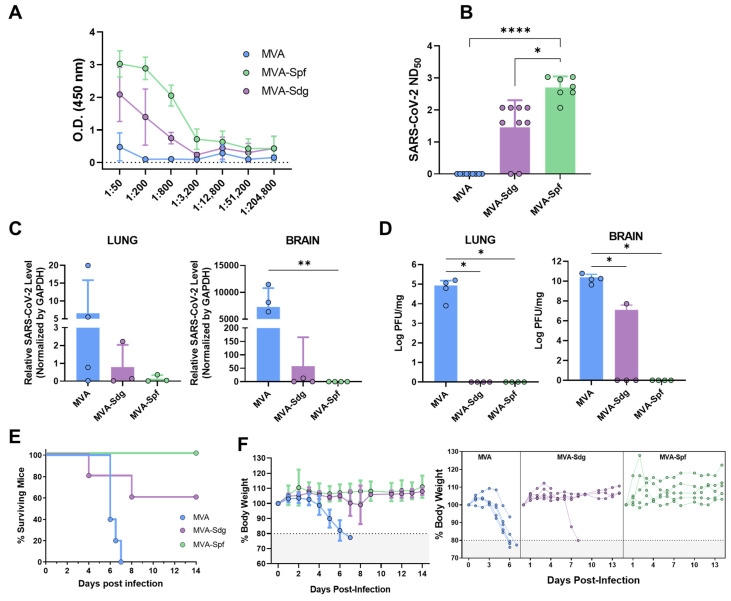
Responses and protection in K18-hACE2 mice immunized with the recombinant MVAs: (**A**) titration of anti-RBD antibodies pre-challenge. Points and bars represent mean values for each group and SD respectively. Both MVA-Sdg and MVA-Spf immunization groups were significantly different from the control group (*p* < 0.05, Kruskal–Wallis test); (**B**) neutralizing antibody titers in sera obtained at 14 days post-boost vaccination. Points represent individual values for each mouse, bars and error bars represent the mean values of each group and SD, respectively. Asterisks denote significant differences between groups by Kruskal–Wallis test (* *p* < 0.0332; **** *p* < 0.0001); (**C**) viral RNA levels detected by quantitative RT-PCR in brain and lung. Points represent mean values for each group and errors bars represent SD. Asterisks denote significant differences between groups (** *p* < 0.0332, Kruskal–Wallis test); (**D**) viral infectious particles in lung and brain tissues after challenge. Points represent mean values for each group and errors bars represent SD. Asterisks denote significant differences between groups (* *p* < 0.05, Kruskal–Wallis test); (**E**) survival after challenge with a lethal dose of SARS-CoV-2. Curves were found to be statistically significant compared with the non-immunized survival curve as calculated by log-rank test (*p* value < 0.05); and (**F**) percentage of body-weight loss caused by SARS-CoV-2 infection as an average within groups (**left**) or individual animals (**right**).

Prior to evaluating the ability of the vaccine candidates to protect against a lethal SAR-CoV-2 infection, we compared the immunogenicity of MVA-Spf or MVA-Sdg in an animal model suitable for testing SARS-CoV-2 protection. To that end, we used transgenic mice expressing hACE2 receptor regulated by the cytokeratin 18 (K18) gene promoter (K18-hACE2). Groups of mice were intramuscularly inoculated with the MVA recombinants following a homologous prime-boost immunization strategy as stated above. Binding antibody titers were measured in sera samples obtained two weeks after boost from immunized animals by ELISA using the purified S-RBD. Both immunized groups had significant levels of specific anti S-RBD antibodies compared with the control group, being significantly higher in MVA-Spf-immunized mice than in the MVA-Sdg-immunized mice (Figure 5A). To test the neutralizing capacity of the anti-SARS-CoV-2 antibodies induced by the vaccine candidates, a PRNT assay was performed using the same serum samples. The NT_50_ values revealed a significant increase of the neutralizing activity in the sera from immunized mice compared to the control group. A significant increase in NT_50_ values was also detected in sera from MVA-Spf-immunized mice compared to the MVA-Sdg-immunized mice (*p* < 0.001) (Figure 5B).

#### 3.6.2. Protection in K18-hACE2 Mice

We next evaluated the ability of the recombinant MVAs to confer protection against SARS-CoV-2 challenge. To that end, K18-hACE2 mice (n = 9) were immunized with the previously described MVA recombinants and subsequently challenged intranasally with 10^5^ PFU of SARS-CoV-2 per mouse at two weeks post-boost. Presence of the virus in brain and lung homogenates was determined in four out of nine immunized animals in each group. To that end, SARS-CoV-2 RNA was measured by qRT-PCR (Figure 5C) and SARS-CoV-2 titers were determined by plaque assay on Vero E6 cells (Figure 5D). High relative expression levels of viral RNA were detected in the lungs and brains of control mice which had been inoculated with MVA. In contrast, infection in MVA-Spf-immunized mice was abolished or under the level of detection by qRT-PCR in all four animals, and only one mouse in the MVA-Sdg immunization group showed viral replication. High levels of infective viral particles were observed in control mice by plaque assay, reaching 10^5^ and 10^10^ PFU per mg of tissue in lung and brain, respectively. Conversely, an absence of infectious viral particles in these organs was confirmed in MVA-Spf-immunized mice. Similarly, MVA-Sdg-vaccinated mice did not present with the infective virus, except for one mouse that displayed a viral titer of 5 × 10^7^ PFU/mg of tissue in brain (the same animal that was positive by qRT-PCR), although this titer was 1000-fold lower than those in the control group.

Survival rates, percentages of body-weight loss and clinical signs were also analyzed and monitored daily two weeks post-challenge in the five remaining mice in each group. By days 6–7 post-infection, control mice lost 20% or more of their initial body weight, showing severe clinical signs of infection. Those that did not succumb to infection were humanely euthanized (Figure 5E,F). Conversely, a partial protection (60% surviving mice) was observed in the group immunized with MVA-Sdg, with only one of the two animals showing signs of infection. Significantly, total protection was achieved in MVA-Spf-immunized mice, as determined by the absence of body-weight loss and clinical signs of infection (Figure S1).

**Figure 6:**
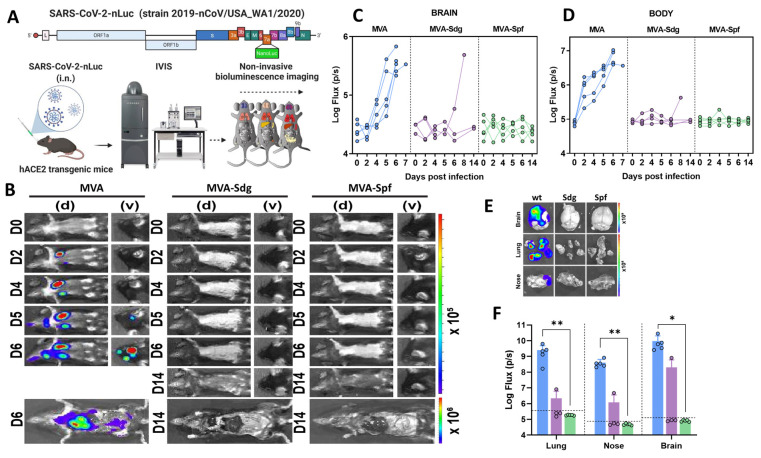
Replication and spread of SARS-CoV-2-WA-nLuc in vaccinated K18-hACE2 mice: (**A**) experimental design to evaluate the in vivo efficacy of MVA vaccine. Animals were infected intranasally, and the infection was monitored using bioluminescence imaging: (**B**) representative BLI images of SARS-CoV-2 WA-nLuc-infected mice in ventral (v) and dorsal (d) position. Scale bars denote flux (photons/s) in log scale; (**C**,**D**) temporal quantification of nLuc signals as flux (photons/s) computed non-invasively. Results for each mouse are shown; (**E**,**F**) ex vivo imaging of isolated organs (**E**) and quantification of nLuc signal as flux (photons/s) in log scale (**F**) after necropsy. Each data point in (**F**) represents the flux value for an individual mouse. Data in (**F**) were analyzed by Kruskal–Wallis test as in legend to Figure 3 (* *p* < 0.05, ** *p* < 0.0332). Images and flux values for day 0 were obtained before challenge. Blue, purple and green colors in (**C**,**D**,**F**) indicate values for MVA, MVA-Sdg and MVA-Spf inoculated mice, respectively.

In parallel, we also employed a BLI to monitor the impact of vaccination on SARS-CoV-2 replication and spread in K18-hACE2 mice using a reporter SARS-CoV-2 expressing nLuc luciferase (Figure 6A) [64]. In control mice, nLuc signal was first detected in the lung at 2 d.p.i. where it continued to increase until 5–6 d.p.i. with infection becoming systemic and reaching the cervical lymph nodes and brain (imaging in ventral position), leading to death (Figure 6B and Figure S2). In contrast, luminescent signals were undetectable in the lungs or brains of MVA-Sdg- or MVA-Spf-immunized animals throughout the course of infection, with the exception of one MVA-Sdg-immunized mouse that succumbed to infection at 8 d.p.i. Interestingly, we observed nLuc signal in the nares of control-immunized mice at 5–6 d.p.i., indicating the presence of transmissible virus which was absent in any of the MVA-Sdg- or MVA-Spf-immunized animals (Figure 6B–D).

In addition, to observe viral spread with higher sensitivity and resolution, body and individual organs were imaged after necropsy. Most organs analyzed from control-vaccinated mice showed nLuc activity, with the highest signal in the brain followed by the lung and nasal cavity (Figure 6E and Figure S3). In contrast, mice immunized with MVA-Spf, which remained healthy throughout the experiment and displayed no signs of disease (including body weight loss phenotype), lacked detectable nLuc activity, lung infection, or neuroinvasion. Among MVA-Sdg-immunized mice, 60% remained protected from infection with undetectable nLuc activity in their organs, and only one mouse showed viral neuroinvasion at 7 d.p.i. and died one day later.

#### 3.6.3. MVA-Spf Controls Cytokine Response in the Brain of SARS-CoV-2 Infected K18-hACE2 Mice

**Figure 7:**
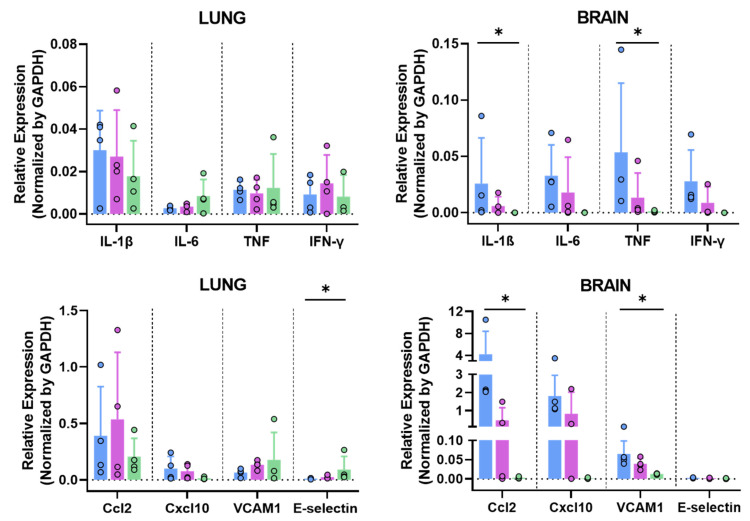
Relative expression of cytokines and chemokines in lung and brain at 6 d.p.i. measured by qRT-PCR. Blue bars, MVA; purple bars, MVA-Sdg; green bars, MVA-Spf. Mouse GAPDH was used as a housekeeping gene. Data were analyzed by Kruskal–Wallis test. *, *p* < 0.05. Blue, purple and green colums correspond to values for MVA, MVA-Sdg and MVA-Spf inoculated mice, respectively.

SARS-CoV-2 infection triggers a hyper-induction of proinflammatory cytokines, also known as cytokine storm or cytokine-release syndrome (CRS), which is one of the key aspects that significantly contributes to the pathogenesis caused by this SARS-CoV-2 infection. To analyze if these MVA-vaccine candidates can prevent this phenomenon, RNA transcripts of different proinflammatory cytokines (Il-1b, Il-6, TNF and IFN-γ) were measured in the brains and lungs at 6 d.p.i. by qRT-PCR. No significant differences were observed in the lungs among groups (Figure 7). Interestingly, a general decrease in the expression levels of these cytokines was observed in the brains of immunized animals, especially in those immunized with MVA-Spf (Figure 7). Additionally, other inflammatory molecules related to the cytokine storm induced by SARS-CoV-2, such as Ccl2 (also involved in neuroinflammatory processes [65]) and Cxcl10, were upregulated in control animals. They also appeared to be upregulated in MVA-Sdg-vaccinated mice, probably indicating SARS-CoV-2 replication. Finally, adhesion molecules, such as VCAM1 and E-selectin, were also upregulated in control mice (Figure 7), possibly indicating vascular injury in this tissue.

### 3.7. Protective Capacity of MVA Vaccines in Golden Syrian Hamsters

**Figure 8:**
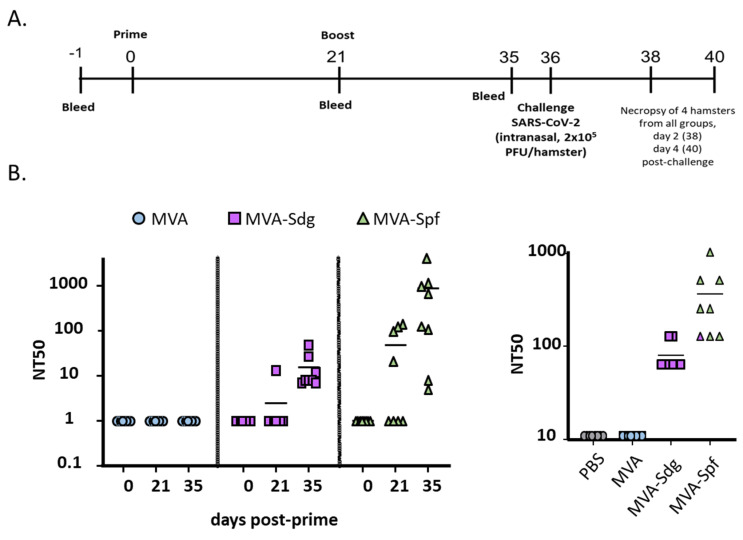
SARS-CoV-2 neutralizing antibodies in sera of vaccinated golden Syrian hamsters: (**A**) timeline of the experiment (days); and (**B**) neutralization titers in sera from hamsters. Neutralization titers were obtained using a VSV pseudotype-based assay (**left**) or infectious SARS-CoV-2 (**right**). Titers in the left panel correspond to day 21 (one inoculation) and 35 (two inoculations), as indicated in the horizontal axis, and those in the right panel correspond to sera from day 35, immediately before challenge.

To further demonstrate the feasibility of using MVA expressing SARS-CoV-2 S as a COVID-19 vaccine, we evaluated the safety, immunogenicity, and protection efficacy in golden Syrian hamsters, which we and others have previously shown to be susceptible to, and represent a good animal model for, SARS-CoV-2 infection, including vaccine development [53,59,60]. To that end, golden Syrian hamsters were either mock-vaccinated with PBS or vaccinated with 10^7^ PFU of MVA, MVA-Sdg, or MVA-Spf using a prime and boost approach (Figure 8A). After vaccination, clinical signs and body weight were measured daily for 21 (prime) and 36 (prime and boost) days. In both cases, hamsters vaccinated with MVA, MAV-Sdg or MVA-Spf did not develop any apparent clinical signs of infection, any changes in body weight, or mortality, demonstrating that vaccination with 10^7^ PFU of MVA, MVA-Sdg, or MVA-Spf is safe. At days −1, 21 (prime) and 35 (prime and boost), serum samples were collected and used to evaluate neutralizing antibody responses using a vesicular stomatitis virus (VSV) pseudotype-based assay or infectious SARS-CoV-2 (Figure 8B). We were able to detect the presence of neutralizing antibodies in the sera of hamsters vaccinated with MVA-Sdg after boost (day 35) while neutralizing antibodies were readily detected by day 21 (prime) in hamsters vaccinated with MVA-Spf. Notably, by day 35 (primer + boost) the levels of neutralizing antibodies were higher than those detected after prime vaccination and higher than those obtained by vaccination with MVA-Sdg (Figure 8B), demonstrating that vaccination with MVA-Spf is able to induce earlier and higher levels of neutralizing antibodies than the MVA-Sdg vaccine. As expected, we were not able to detect SARS-CoV-2 neutralizing antibodies in hamsters mock (PBS)-vaccinated or vaccinated with MVA, on any day post-vaccination.

**Figure 9:**
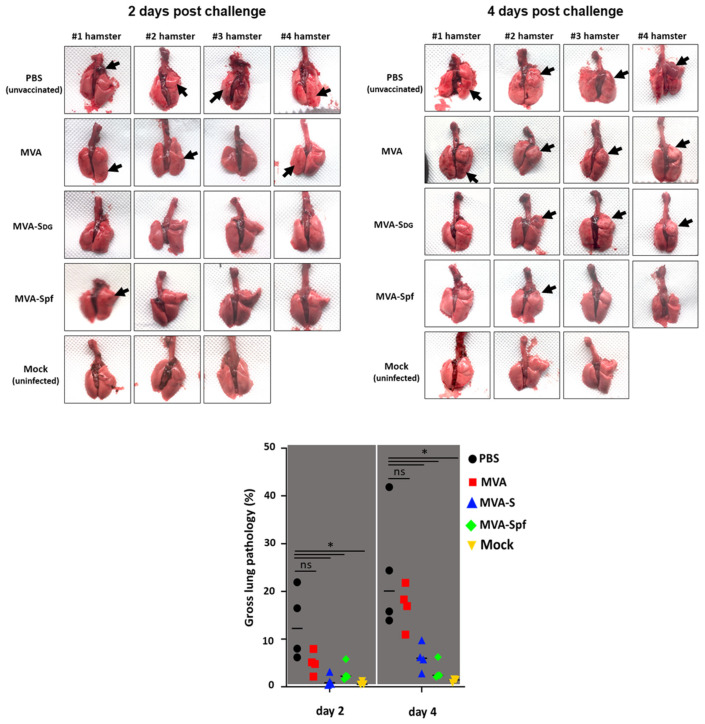
Protection from lung pathology in the hamster model. Images of the lungs from hamsters on days 2 and 4 after challenge with SARS-CoV-2 are shown. # denote the animal number within each group. Note the complete absence of red lesions (arrows) in animals vaccinated with MVA-Spf. Lower panel shows the gross lung pathology score of the animals in each of the experimental groups. *p* values for the difference between the PBS-treated group and experimental groups were estimated by two-tailed *t* test, * = *p* < 0.0055. ns, not significant.

To assess protection efficacy, vaccinated hamsters were next challenged with 2 × 10^5^ PFU of SARS-CoV-2, and nasal turbinate and lungs were collected at days 2 and 4 after challenge to assess the presence of virus, and to evaluate pathology in the lungs. Mock (PBS)-vaccinated hamsters challenged with SARS-CoV-2, and unvaccinated and mock-challenged hamsters were included as internal controls. As expected, lungs from unvaccinated hamsters challenged with SARS-CoV-2 showed pathology lesions at both days post-challenge but were higher on day 4 than on day 2 after SARS-CoV-2 challenge (Figure 9). Similar pathology lesions were observed in the lungs of hamsters vaccinated with MVA. Notably, pathology lesions were significantly lower in hamsters vaccinated with MVA-Sdg or MVA-Spf on days 2 and 4 post-challenge as compared to mock- or MVA-vaccinated hamsters. As expected, we did not observe any pathology lesions in the lungs of hamsters that were not challenged with SARS-CoV-2 on either day 2 or 4 post-challenge.

**Figure 10:**
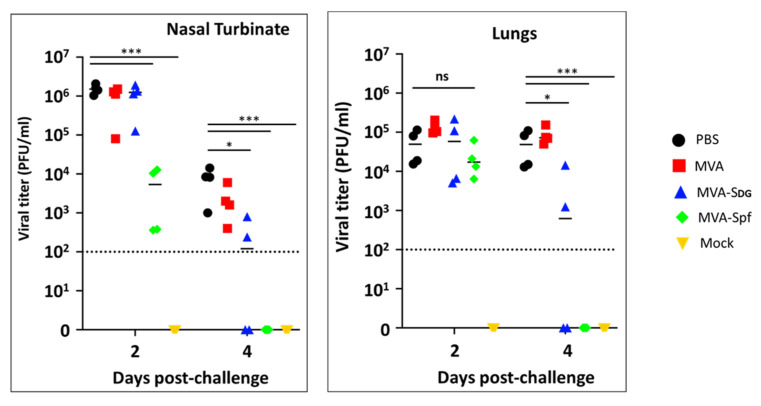
Protection in the hamster infection model. SARS-CoV-2 viral titers at 2 and 4 days after challenge were determined by plaque assay. Dotted line indicates the detection limits in our assay. Values below the limit are plotted as 0. Statistical analysis by 2-tailed *t* test, * = *p* < 0.0055, and *** = *p* < 0.000055. ns, not significant.

Notably, these pathology results correlated with the viral titers in the nasal turbinate and lungs (Figure 10). Higher levels of SARS-CoV-2 were detected on both days 2 and 4 and in the nasal turbinate and lungs of hamster mock (PBS)-vaccinated hamster. Similarly, high levels of SARS-CoV-2 replication were also observed in the nasal turbinate and lungs of hamsters vaccinated with MVA on days 2 and 4 after challenge with SARS-CoV-2. Levels of SARS-CoV-2 in the nasal turbinate and lungs of hamsters vaccinated with MVA-Sdg on both days after challenge with SARS-CoV-2 were significantly lower than those of PBS- or MVA-vaccinated hamsters. Likewise, significantly lower levels of SARS-CoV-2 were detected in the nasal turbinate and lungs of hamsters vaccinated with MVA-Spf on either day 2 or 4 post-infection. Notably, in the case of hamsters vaccinated with MVA-Spf, we were not able to detect the presence of SARS-CoV-2 in the nasal turbinate or lungs on day 4 post-challenge, indicating a high degree of protection both in the central nervous and respiratory systems. These results demonstrate that vaccination with MVA-Sdg, and more notably with MVA-Spf, can protect hamster from SARS-CoV-2 infection, with lower levels of viral replication in the upper (nasal turbinate) or lower (lung) respiratory tract, as compared to unvaccinated animals.

## 4. Discussion

The recent COVID-19 pandemic has demonstrated the usefulness of vaccines to control the spread of infectious diseases and has reminded us of the importance of updating and improving vaccine technologies. In addition to the approved vaccines based on messenger RNA and non-replicating adenoviruses, other technologies offer specific advantages that may expand our tool plethora for vaccination strategies. Among the relevant aspects to consider in choosing vaccine platforms are the cost, thermal stability and ease of administration, and the optimal characteristics of vaccines in particular circumstances should be sought. In this respect, vaccine candidates that provide different advantages with respect to those already approved are of interest.

In this study, we took advantage of an efficient MVA cloning/selection system to isolate MVA recombinants expressing SARS-CoV-2 S glycoprotein to test them as COVID-19 vaccine candidates. In preclinical testing, we demonstrated that those candidates were suitable for achieving protection from SARS-CoV-2-induced pathogenicity in both mice and hamsters, as has been noted with MVA recombinants obtained with different procedures [39,40,41,46,66]. Even though unmodified S protein has been shown to induce protection, we showed that a prefusion-stabilized version (Spf) is clearly superior in the induction of immunity, including neutralizing antibodies, and in protection from disease, in agreement with previous works [43,45,47]. Our study complements other previously published research in which MVA recombinants were tested on animals. We have used some specific strategies and methodologies, such as a different cloning/vector system and the use of an nLuc-expressing virus to facilitate SARS-CoV-2 distribution in the whole bodies of infected animals. Overall, the results obtained by vaccination with MVA recombinants expressing the SARS-CoV-2 S protein rendered a positive vaccine profile, with MVA-Spf providing effective protection against SARS-CoV-2-induced pathogenesis in both mice and hamsters. This included reducing lesions in the lungs, a major site of virus replication and a crucial factor in COVID-19 pathogenesis. In addition to the induction of immunity, a reduction in viral RNA and virus titers in the respiratory tract was detected. Also, we have shown a complete absence of virus replication in the brains of animals vaccinated with MVA-Spf. However, MVA-Sdg only partially decreased neuroinvasion. Thus, it seems likely that vaccine potency is crucial to preventing the spread of the SARS-CoV-2 infection to the brain. In this respect, several vaccination strategies have resulted in varied levels of brain protection [51,67,68], which is a significant factor due to the likely relationship with COVID-19 neurological consequences.

With the ongoing COVID-19 pandemic, numerous candidate vaccines are being investigated at an unprecedented pace. Among the hefty experience gained during the pandemic, the results presented in this study support further development of the MVA-Spf vaccine and hopefully add to our current understanding and knowledge base for the improvement of poxvirus-based vaccines.

## 5. Conclusions

It is well-established that vaccines exposing the SARS-CoV-2 S protein to the immune system can provide good protection against severe disease. Our work, in conjunction with recent publications, reinforces the idea that recombinant MVA vaccines are potent immunogens that can complement other vaccine technologies. Our experimental data demonstrates that this is indeed the case with recombinant vaccinia MVA virus expressing the full-length S protein. Furthermore, our findings indicate that prefusion-stabilized SARS-CoV-2 S can significantly improve the level of protection against clinical signs of disease, as well as enhance neutralizing antibody responses and provide protection in the brain.

## Data Availability

The data presented in this study are available on request from the corresponding authors.

## Supplementary Materials

The following supporting information can be downloaded at: https://www.mdpi.com/article/10.3390/vaccines11051006/s1, Figure S1: Pathological evaluation of K18-hACE2 transgenic mice vaccinated and challenged with SARS-CoV-2-WanLuc, Figure S2: BLI images of SARS-CoV-2 WA-nLuc infected mice in ventral (v) and dorsal (d) position at indicated days post infection, Figure S3: Ex vivo imaging of whole body and organs (brain, nose and lung) at indicated days after necropsy.
