# Supplementary figures and images for: Vaccinia Virus Strain MVA Expressing a Prefusion-Stabilized SARS-CoV-2 Spike Glycoprotein Induces Robust Protection and Prevents Brain Infection in Mouse and Hamster Models
Source: Vaccines (Basel). 2023 May 21;11(5):1006. doi: 10.3390/vaccines11051006 (PMC10220993; doi:10.3390/vaccines11051006)

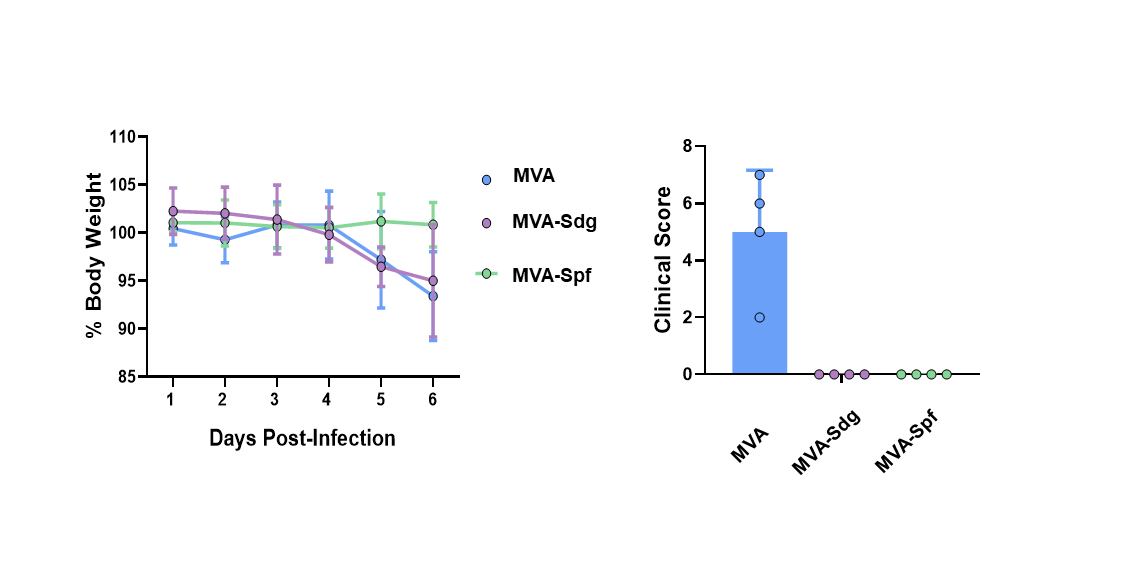

Supplement: Supplementary file 1 [file vaccines-11-01006-s001.zip › Figure S1.tif]

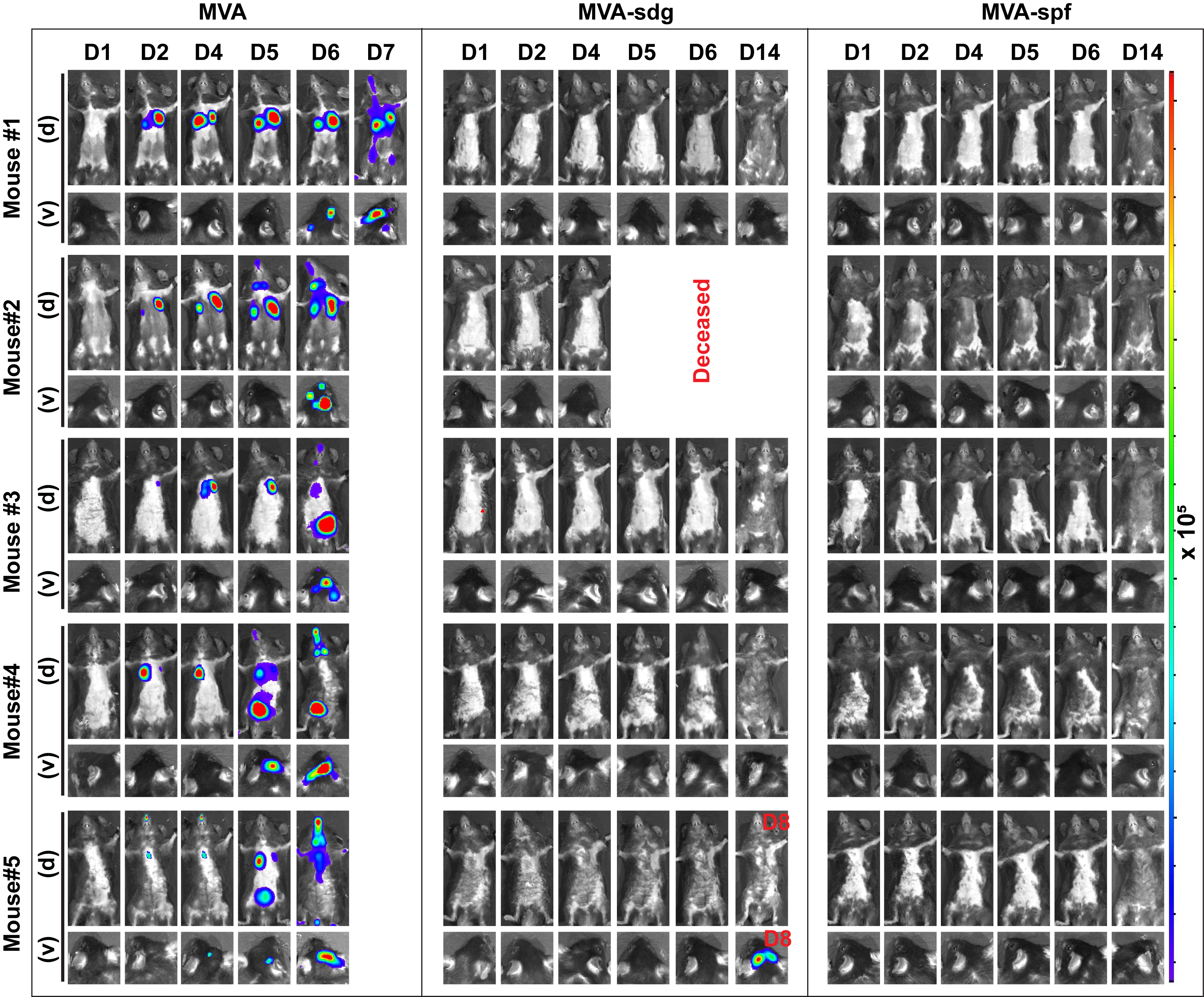

Supplement: Supplementary file 1 [file vaccines-11-01006-s001.zip › Figure S2.tif]

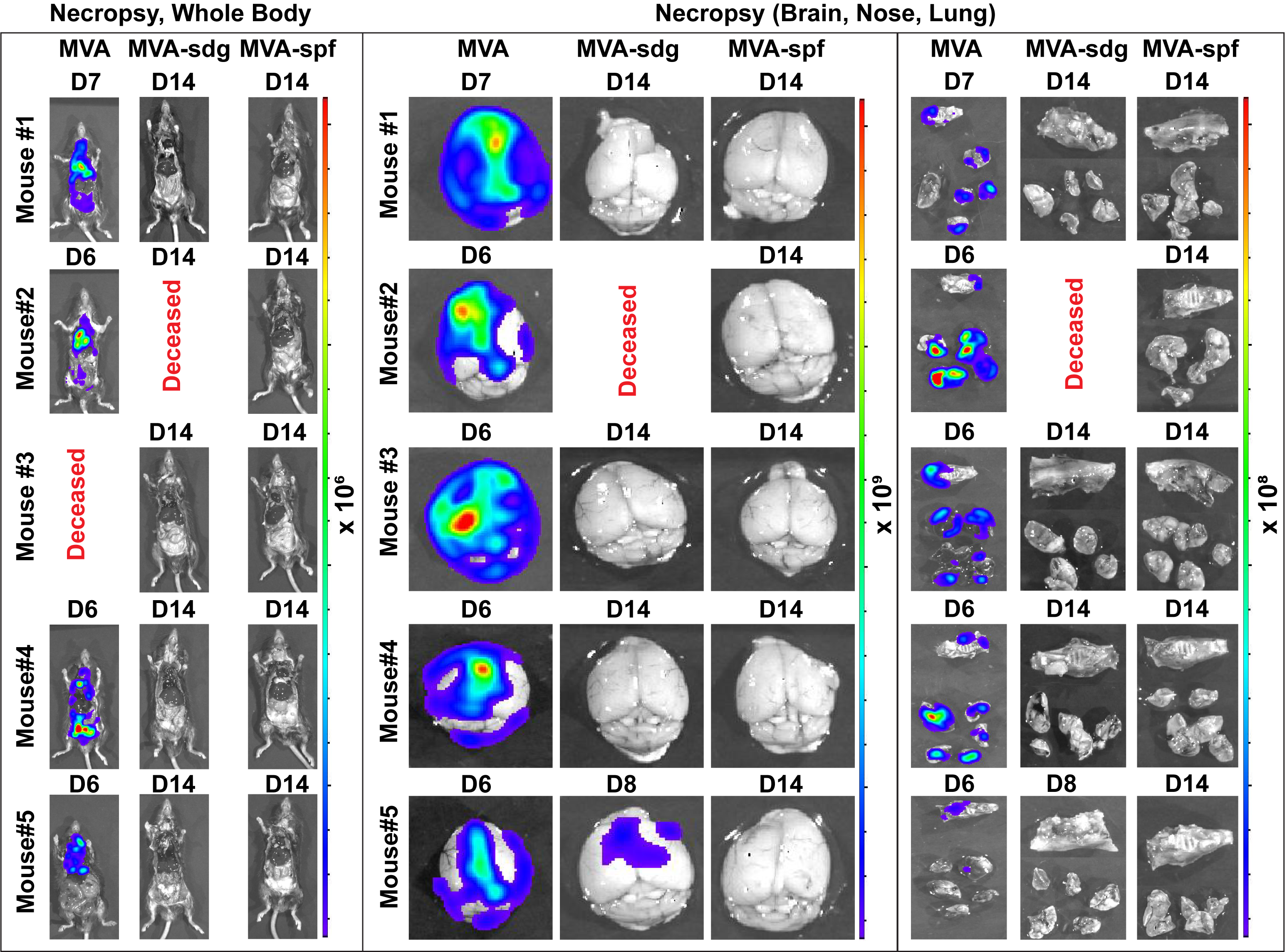

Supplement: Supplementary file 1 [file vaccines-11-01006-s001.zip › Figure S3.tif]
